# Considerations and caveats for analyzing chromatin compartments

**DOI:** 10.3389/fmolb.2023.1168562

**Published:** 2023-04-05

**Authors:** Achyuth Kalluchi, Hannah L. Harris, Timothy E. Reznicek, M. Jordan Rowley

**Affiliations:** Department of Genetics, Cell Biology and Anatomy, University of Nebraska Medical Center, Omaha, NE, United States

**Keywords:** compartments, Hi-C, Micro-C, chromatin organization, eigenvector, subcompartment

## Abstract

Genomes are organized into nuclear compartments, separating active from inactive chromatin. Chromatin compartments are readily visible in a large number of species by experiments that map chromatin conformation genome-wide. When analyzing these maps, a common step is the identification of genomic intervals that interact within A (active) and B (inactive) compartments. It has also become increasingly common to identify and analyze subcompartments. We review different strategies to identify A/B and subcompartment intervals, including a discussion of various machine-learning approaches to predict these features. We then discuss the strengths and limitations of current strategies and examine how these aspects of analysis may have impacted our understanding of chromatin compartments.

## 1 Introduction

**FIGURE 1:**
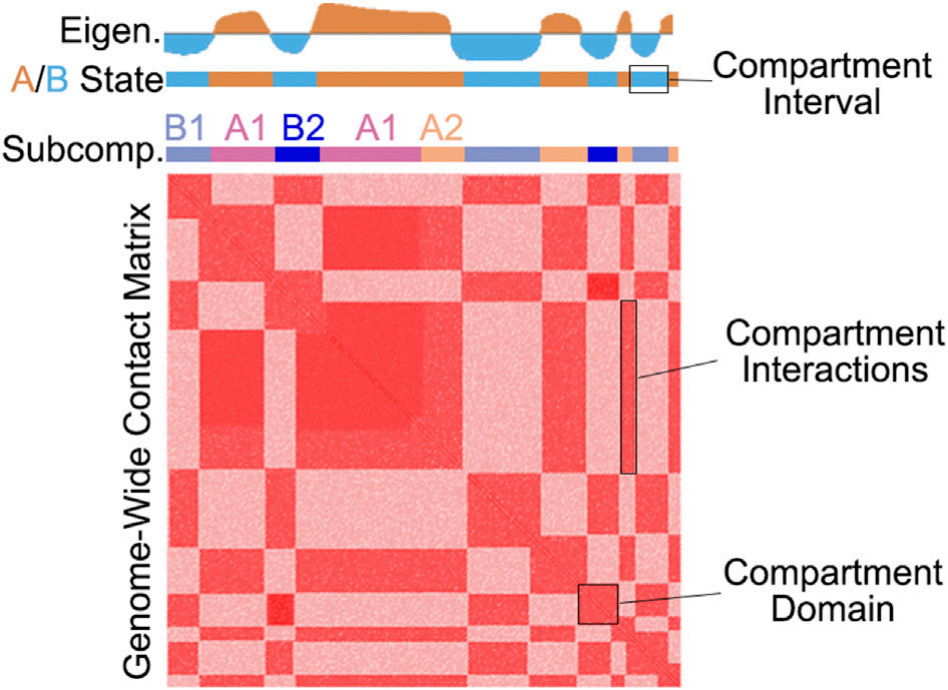
An illustration of the checkerboard pattern commonly found by whole-genome chromatin conformation assays such as Hi-C and Micro-C. The top tracks illustrate an example eigenvector as well as A/B and subcompartment classification.

Genomic DNA is organized into intricately folded structures, providing the context for storing and accessing genetic information. Thanks to 3D genome folding, loci separated by hundreds of kilobases of sequence can influence each other *via* long-range chromatin-chromatin interactions (Rowley and Corces, 2018; Fulco et al., 2019). Chromatin organization can drastically differ between cell types, in response to stimuli, during differentiation, and due to disease, indicative of a highly responsive and functionally important nuclear environment (Shlyueva et al., 2014; Dixon et al., 2015; Chakraborty and Ay, 2019; Lu et al., 2020; Winick-Ng et al., 2021; Rocks et al., 2022). Genomes are organized into several distinct architectural features which can be measured by various high-throughput sequencing approaches (Rowley and Corces, 2018), including both Hi-C and Micro-C which measure chromatin conformation genome-wide (Lieberman-Aiden et al., 2009; Rao et al., 2014; Hsieh et al., 2015; Hsieh et al., 2020; Krietenstein et al., 2020). These maps display a distinctive plaid-like pattern indicative of the physical segregation of active and inactive chromatin into compartments (Lieberman-Aiden et al., 2009) (Figure 1).

The term “compartment” refers to a group of loci that preferentially interact with each other, likely because of similarities in chromatin activity status (Rowley and Corces, 2018). While the term “compartment” refers to the biological phenomenon, chromatin contacts that comprise compartments can be referred to as compartmental interactions. To differentiate these 3D and 2D features from the 1D genomic loci that make up these features, we can refer to a stretch of loci within the same compartment as a compartment interval. Importantly, compartment interactions are not restricted to long-range, and compartment intervals form a domain-like pattern, i.e., a triangle in Hi-C, which we refer to as compartment domains (Rao et al., 2017; Rowley et al., 2017) (Figure 1).

While not the focus of this review, we should mention that several other features can make domain-like structures, including CTCF loop domains as well as intervals that are excluded from loops, termed “ordinary” domains (Rao et al., 2017). Therefore, domains that are identified by chromatin conformation assays can be composed of multiple distinct organizational principles. Others have discussed the relationship between these distinct features and Topologically Associated Domains (TADs) (Szabo et al., 2019; Beagan and Phillips-Cremins, 2020).

Chromatin compartments are relatively ubiquitous features of genome organization, having been detected in Hi-C maps across many of the tested eukaryotic phyla as well as in archaeal species (Dong et al., 2017; Rowley et al., 2017; Takemata et al., 2019). Despite their prevalence, mechanisms explaining the formation, regulation, and function of compartments remain somewhat mysterious. By discussing current methods of compartment analysis, we shed light on limitations that may contribute to the debate concerning the biological nature and responsiveness of compartmental features.

## 2 Identification of A/B compartments

### 2.1 Eigenvector (PCA-based approach)

A plaid-like pattern was evident from the first Hi-C map published, denoting the broad separation of activity states into A and B compartments (Lieberman-Aiden et al., 2009). To assign genomic intervals to these two segregating states, the authors obtained the leading eigenvector from Principal Component Analysis (PCA) on the Hi-C contact matrix. The leading eigenvector represents a continuous signal along the genome with both positive and negative values serving to categorize loci as either A or B compartment intervals (Figure 1). This eigenvector-based approach has been the predominant method to identify A and B compartment intervals, and many different Hi-C data analysis tools implement PCA for compartment interval identification (Durand et al., 2016; Giorgetti et al., 2016; Kruse et al., 2020; van der Weide et al., 2021). These tools, however, sometimes differ in data preparation steps that occur before the calculation of the eigenvector, and it is not clear how much, if at all, these subtle differences impact the results.

#### 2.1.1 Visibility correction

**FIGURE 2:**
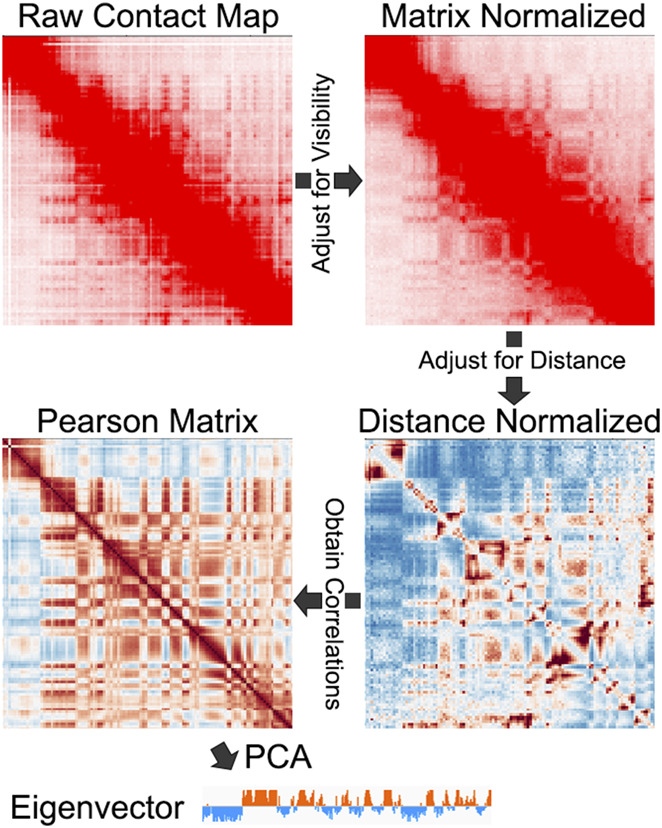
An example of the steps involved in A/B compartment identification, including matrix balancing, distance normalization, Pearson correlation, and PCA. Contact Map from Rowley et al., 2017.

A common initial step is to normalize the map to account for the “visibility” of each bin (Figure 2). The rationale for this step is to help account for digestion preferences, locus mappability, GC content, and other known and unknown influences (Yaffe and Tanay, 2011; Imakaev et al., 2012; Rao et al., 2014; Servant et al., 2015). Several different normalization schemes are available for visibility correction, examples of which include probabilistic models (Yaffe and Tanay, 2011), iterative correction and eigenvector decomposition (ICE) (Imakaev et al., 2012), Knight-Ruiz (KR) matrix balancing (Knight and Daniel, 2013; Rao et al., 2014), among others. Interestingly, one study compared various normalization methods and found advantages and disadvantages (Lyu et al., 2020). However, it is not clear how these different methods specifically impact compartment identification.

#### 2.1.2 Distance decay

Genome-wide maps of chromatin contacts display a decay of interaction signal in that the frequency of interactions decreases with genomic distance (Lieberman-Aiden et al., 2009). While this diagonal decay likely reflects the physical properties of chromatin (Sanborn et al., 2015), it means that Hi-C and Micro-C signals are low and/or sparse at long distances. Typically, PCA-based compartment identification considers the whole chromosome, making it essential to account for distance-based effects. Therefore, in addition to locus visibility adjustment, compartment identification methods often implement distance-based normalization (Figure 2). This can be done by dividing the signal at each bin-pair (i.e., observed) by the average signal at that distance (i.e., expected): $\frac{Observed}{Expected}$ (Durand et al., 2016). While frequently used, there are alternate distance normalization methods. For example, we often use $\frac{Observed+1}{Expected+1}$ to help mitigate long-distance value inflation that can occur when the expected value drop too low (Rowley et al., 2020). Others account for distance effects by loess normalization (Giorgetti et al., 2016). Because of the widespread use, but varying methods of distance normalization, it will be valuable to explore alternatives and their impact on compartment identification.

#### 2.1.3 Bin-to-bin correlation

Many algorithms use the visibility corrected, and distance normalized values to then create Pearson correlation matrices (Figure 2). The intensity within this matrix no longer represents contact strength, but rather represents the Pearson correlation coefficients between each pair of genomics bins. Essentially this matrix describes the similarity between locus_x_ and locus_y_ when considering their signal patterns across the entire chromosome. While this can relate to signal strength to some degree, it is possible to derive high correlation values for highly similar loci that do not have high-intensity interactions. Therefore, the eigenvector is not a measure of compartment interaction strength. Instead, the eigenvector typically reflects locus correlations.

The above steps represent a general workflow commonly employed to prepare contact matrices for PCA. We described this workflow to highlight the large number of processing steps that typically occur before eigenvector calculation. However, available compartment identification tools vary, and it is not clear how differences in each step may alter the compartment calls at various resolutions. In the future, a detailed analysis of the impact of each step would be informative.

### 2.2 Limitations of compartment identification by PCA

**FIGURE 3:**
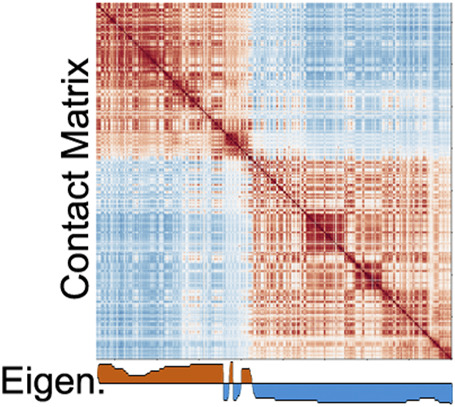
Illustration of how other organizational features may be detected by the leading eigenvector instead of A/B compartments. Contact map from Rowley et al., 2017.

While PCA for compartment analysis is a common and valuable approach, users should be aware of some limitations. The first eigenvector represents the principal component with the largest variance, which may or may not represent genomic compartment segregation. Indeed, in some Hi-C maps the first principal component reflects other prominent features. For example, Hi-C maps in *D. melanogaster* display a plaid compartment pattern within each chromosome arm, but interactions that span the arms are exceptionally weak. When run on the whole chromosome, the leading eigenvector reflects the separation of arms instead of compartments (Hou et al., 2012; Sexton et al., 2012; Rowley et al., 2017) (Figure 3). Therefore, the first principal component may not always depict the plaid A/B pattern, even when there is an apparent plaid-like pattern within the segments. It is possible that the second principal component could be used in such cases. However, because the eigenvector is derived on a per-chromosome basis, the results of each chromosome must be inspected to ensure each represents the plaid pattern.

One secondary and minor drawback is that A/B compartments are typically assigned to each chromosome, or sometimes each arm, independently. Because the initial sign of the eigenvector is somewhat arbitrary, the resultant profile must be examined on a per-chromosome basis and overlapped with active chromatin activity states, genes, or GC content. Indeed, a decision of whether or not to invert the eigenvector is made per chromosome to ensure that positive and negative values consistently correspond to features within the A and B compartments, respectively (Durand et al., 2016; Giorgetti et al., 2016; Kruse et al., 2020; van der Weide et al., 2021). While this is typically viewed as a minor inconvenience, assigning positive/negative signs for each chromosome could lead to potential mistakes, especially considering experiments that might impact the relationship between compartments and chromatin marks.

**FIGURE 4:**
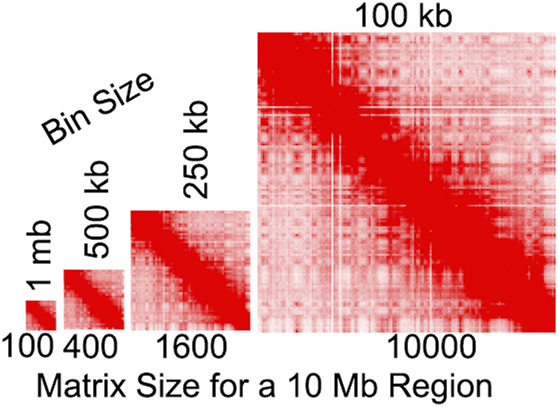
Illustration of the relationship between bin size and matrix size. Heatmap sizes are proportional to the number of bins. Contact map from Rao et al., 2014.

Our recent work suggests that bin size should be a major consideration, as it can impact the results of compartment identification (Rowley et al., 2017; Gu et al., 2021). Below, we discuss the impact of bin sizes on our understanding of compartments, but there are a few PCA-relevant considerations. In Hi-C and similar whole-genome methods, data binning is often necessary to ensure adequate signal, particularly at long distances. Obtaining a sufficiently deep signal across the entire chromosome is essential for PCA to derive states. In the first published Hi-C map, data was binned at 1 Mb, a bin size that has since been often used for A/B compartment identification (Lieberman-Aiden et al., 2009). However, methodological and technological advancements are making it possible to achieve finer scales which continues to revise our understanding of these organizational features (Gu et al., 2021; Goel et al., 2022). Yet this refinement brings its own challenges. For PCA-based analysis, fine-scale binning can lead to extensive memory and computation time requirements for data in dense matrix format (Gu et al., 2021). These requirements become increasingly prohibitive with finer scales. For example, a 10-fold change in resolution leads to a 100-fold larger matrix (Figure 4). Indeed, creating a dense matrix of human chromosome 1 at 1 kb would have c.a. 62 billion entries and require an estimated nearly 500 GB of RAM just to read the dense matrix into memory. As sequencing costs lower and with the development of fine-scale methods such as Micro-C (Hsieh et al., 2015; Hsieh et al., 2020; Krietenstein et al., 2020), algorithms will need to find ways to perform memory-efficient fine-scale compartment analysis.

### 2.3 POSSUMM (PCA of sparse, super massive matrices)

To overcome computational limitations, POSSUMM calculates the eigenvector from massive matrices in sparse format. This algorithm, combined with high sequencing depth, enabled our recent high-resolution compartment identification (Gu et al., 2021). POSSUMM combines matrix-vector product calculation with the power method to enable the computation of principal components without the need to perform calculations on the dense matrix. Due to these features, POSSUMM was recently able to identify compartment intervals in human lymphoblastoid cell lines at 500 bp resolution within minutes using minimal RAM (Gu et al., 2021). This resolution of compartment identification enabled the discovery of multiple new insights regarding the size and nature of compartments. Notably, fine-scale compartment identification at this extremely fine-scale resolution was only possible thanks to the unprecedented sequencing depth within the map, 33 billion contacts. Thus, even with solving computation considerations, eigenvector-based compartment identification at exceptionally high resolution is still unachievable with the sequencing depths obtained by the majority of currently published maps. However, other methods, such as reported in the new preprint on Region Capture Micro-C, have potential to detect fine-scale compartmental features within specified regions (Goel et al., 2022).

### 2.4 Alternative methods of A/B compartment identification

While searching for tools that identify A/B compartment intervals from Hi-C maps, we noticed that most studies use an eigenvector-based approach. As an alternative, CscoreTool is a compartment identification tool that calculates the probability of each bin being in the A compartment (Zheng and Zheng, 2018). This method defines compartments by considering and learning parameters including distance-based effects, Hi-C experimental bias factors, and compartmental probability scores. These parameters are randomly initialized, followed by iterative refinement using maximum-likelihood estimation. CscoreTool was able to report compartment intervals in 1 kb bins, which were not obtainable by the dense-matrix PCA approach. At other resolutions, CscoreTool had reportedly lower memory and time requirements than dense-matrix PCA but still required nearly 3 days to calculate compartment intervals in 1 kb bins. The compartment intervals identified by CscoreTool and eigenvector were similar, yet not 100% identical. Indeed, correlation with accessibility indicated that CscoreTool could be more reflective of the chromatin accessibility state. Thus, while a PCA-based method is the most used, the strengths and weaknesses of other methods should be considered. During the innovation and testing of new algorithms, it will be valuable to compare to current methods without equating the eigenvector-based compartments as ground truth. The innovation of compartment algorithms will provide alternative perspectives and ensure rigor in detecting and analyzing such prominent features.

## 3 Subcompartment identification

### 3.1 Unsupervised clustering

Several groups have proposed that a two-state A/B compartment model may be insufficient to reflect compartmental patterns accurately. Indeed, in 2011, k-means clustering was used to assign interactions to 3-states (Yaffe and Tanay, 2011). In 2014, *in-situ* Hi-C allowed further categorization into subcompartments, predominately done at 100 kb resolution (Rao et al., 2014). Using unsupervised clustering methods, Hidden Markov Model (HMM), K-means, and Hierarchical, the authors noted that chromatin can segregate into at least six subcompartments. Two were denoted as A (A1, A2) while four as B (B1, B2, B3, B4).

Like PCA, subcompartment identification required several data preparation steps, including read binning, matrix balancing, removal of low coverage rows and columns, and z-score calculation. As with any approach, there are several limitations. For example, k-means partitions loci into a user-defined number of clusters. Rao et al. explored several values, noting that 4–8 clusters matched the visible pattern in their dataset. However, it is unclear whether this number of clusters is always suitable for maps of cells under different conditions, in different cell types, or in other organisms. It will be valuable to examine the appropriate number of clusters for each scenario.

### 3.2 Tools for subcompartment identification

Many tools have been several recently developed for subcompartment identification. These differ in methodology, tested bin sizes, and the number of identified subcompartment states. We highlight a few aspects of some of these tools.

#### 3.2.1 Subcompartments from interchromosomal contacts

Subcompartment iNference using Imputed Probabilistic ExpRessions (SNIPER) employs a neural network and denoising autoencoder with multi-layer perceptron (MLP) to impute inter-chromosomal interactions and categorize loci into subcompartments (Xiong and Ma, 2019). SNIPER produces low-dimensional latent variables to classify genomic intervals into one of five primary subcompartment classes—A1, A2, B1, B2, and B3. *SCI:* Sub-Compartment Identifier (SCI) uses graph embedding and k-means clustering on inter-chromosomal interactions (Ashoor et al., 2020). Using gap statistics, SCI determined that nine clusters were optimal but noted that a few of the clusters had similar chromatin marks.

#### 3.2.2 Subcompartments from intrachromosomal contacts

In contrast to the above methods, Calder looks at short-range intra-chromosomal interactions to classify domains into a compartmental hierarchy (Liu et al., 2021). The creators of Calder report on eight subcompartments at 10 kb resolution, but the use of hierarchical clustering allows one to adjust the number of subcompartments. In theory, each node of the dendrogram could be used to define a separate subcompartment. *MOSAIC*: The first eigenvector is often used to identify A/B compartment states, but a recent study found that the other eigenvectors can be used for subcompartment annotation (Wen et al., 2022). Modularity and Singular vAlue decomposition-based Identification of Compartments (MOSAIC) uses intrachromosomal interactions to derive the first two eigenvectors. These eigenvectors are then assigned as subcompartments through k-means clustering. This method identifies four optimal clusters and provides increased concordance in cell-type specificity of subcompartments and gene expression.

These examples highlight the diversity of employed subcompartment identification strategies. It is remarkable, therefore, that the eigenvector-based strategy predominates A/B compartment identification. Subcompartment strategies differ on many aspects, including bin-size and the number of identified subcompartments. A major difference is the use of interchromosomal v.s. intrachromosomal interactions. While using interchromosomal interactions can help interference from other types of architectural features, interchromosomal interactions are sparse thanks to the partitioning of loci into chromosome territories. In contrast, methods that use intrachromosomal maps take advantage of the higher signal but must include strategies that can account for other prominent interaction features. In considering these differences, it is remarkable that compartmental interactions span between separate chromosomes, despite territories. It would be interesting to define a potential differential impact of chromosome territories on subcompartments by comparing these features inter-v.s. intra-chromosomally.

## 4 Compartment prediction

Highly related to A/B compartment and subcompartment identification, many groups have made progress in predicting these features. Noting the relationship between compartments and chromatin marks, a 2015 report predicted A/B compartment intervals from a bin-to-bin correlation between DNA methylation or DNAse hypersensitivity (Fortin and Hansen, 2015). Using this genomic feature correlation method in 100 kb bins, they obtained approximately 0.70–0.89 correlation with the eigenvector. Similarly, we previously predicted compartmental interactions from a bin-to-bin correlation of transcription (e.g., from Global Run On sequencing, GRO-seq). We used this method to predict the 2D compartmental interaction pattern in *D. melanogaster* at 5 kb (Rowley et al., 2017)*.* While GRO-seq alone performed well (*R* = 0.82), simulating insulation by incorporating insulator protein ChIP-seq data improved the correlation (*R* = 0.91). This simple correlation-based method also helped demonstrate that similar principles of compartmental organization exist in a species of worms, plants, and fungi. These basic methods also reveal that the 1D chromatin activity status can predict 2D A/B compartments.

The application of machine learning further improves the prediction of the 3D genome. For example, a convolutional neural network (CNN) trained on compartment annotations and the reference genome sequence predicted A/B compartment intervals with ∼80% accuracy at 100 kb (Kirchhof et al., 2021). That is quite a remarkable feat given that the method solely considers the genomic sequence as input (Kirchhof et al., 2021).

While it is intriguing how well genomic sequence can predict compartments, it is generally accepted that compartments vary between cell types (Kim et al., 2020; Nichols and Corces, 2021; Chakraborty et al., 2022). Implementing cell-type specific signals, a preprint article describes CoRNN, which uses histone modification ChIP-seq data with recurrent neural networks to predict chromosome compartments at 100 kb (Zheng et al., 2022). This strategy improves the accuracy of predictions for compartmental intervals that differ between cell types. Interestingly, while a combination of histone modifications worked best, H3K27ac and H3K36me3 were the most relevant for accurate A/B prediction. Another example, MEGABASE + MiChroM, relates ChIP-seq data with compartments to infer chromatin structural types using neural networks (Di Pierro et al., 2017). These chromatin types are then used within an energy landscape model to predict compartmental interactions at 50 kb. In contrast to the above methods, this neural network approach found that, while H3K27ac is high in the A compartment, it is a poor predictor within this method. Instead, a combination of histone modifications and nuclear proteins served as a better predictor. Indeed, somewhat distinct from the A/B and “sub’compartment models, different combinations of chromatin marks may create distinct compartments. For example, H3K9me3 may actually represent a third compartment, seen by strong interactions with other H3K9me3 sites, and generally weak interactions with A or B compartment intervals (Nichols and Corces, 2021). Indeed regression-based machine learning found that H3K27ac, H3K27me3, and H3K9me3, as well as the absence of all three, were the best indicators of multi-state compartments, and demonstrated an ability to simulate the compartmental pattern at 100 kb using attraction-repulsion maps (Nichols and Corces, 2021).

Many of the above compartment prediction strategies used 50–100 kb bins. However, it is becoming increasingly clear that compartment intervals are smaller than previously supposed (Rowley and Corces, 2018; Gu et al., 2021). It will be interesting to test the effectiveness of these methods when predicting small compartment intervals. To do this, computational efficiency, and the ability to validate higher-resolution predictions in a cell-type specific manner will be important considerations. Additionally, the above prediction methods are almost always evaluated relative to eigenvector-based compartment calls. Because the eigenvector method of defining compartments has its own limitations, it would be of value to reexamine these algorithms with alternative compartment identification strategies.

In addition to these strategies focused on compartment prediction, we wish to note that there are several methods that are designed to predict signal *de novo* or to enhance low-depth signal within 2D contact maps (Zhang et al., 2018; Carron et al., 2019; Liu et al., 2019; Liu and Wang, 2019; Schwessinger et al., 2020; Cheng et al., 2021; Tan et al., 2023). These methods often use neural networks and are now demonstrating remarkable accuracy. It will be interesting to use these methods to learn more about fine-scale compartments and to compare to high-resolution Hi-C/Micro-C maps.

## 5 Potential limitations in compartment analysis

### 5.1 Bin size/resolution

**FIGURE 5:**
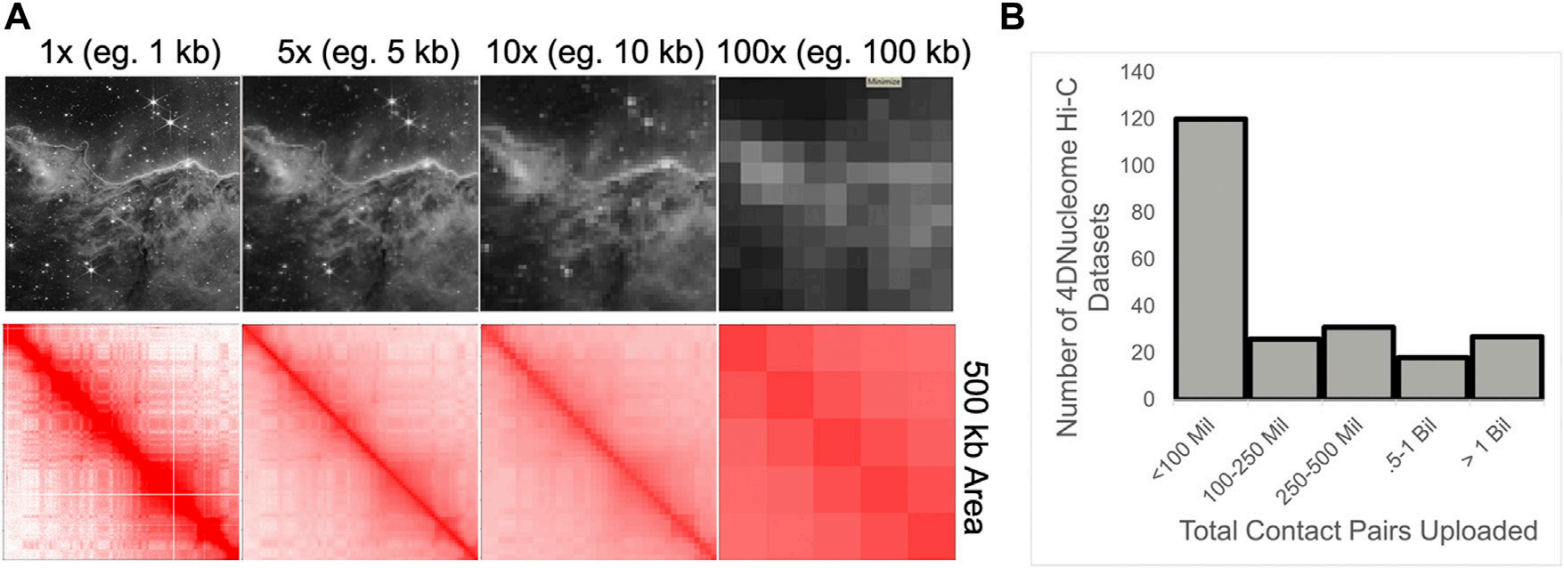
**(A)** Demonstration of how data binning results in the lost ability to detect smaller features. Bottom: Binning of Hi-C data within a 500 kb region. Contact map from Rao et al., 2014. Top: The same binning of an image depicting the Carina Nebula from the James Webb Space Telescope; Credit: NASA. **(B)** Histogram of the reported number of combined contact pairs for Hi-C experiments uploaded to the 4DNucleome database.

As mentioned above, chromatin interaction map analysis often includes data binning into large 2D matrices, where each bin represents the sum of read-pairs connecting two genomic intervals. While this strategy reduces sparsity, data binning blurs distinct components. Indeed, large bins make it difficult to detect small patterns (Figure 5A). Previously, we speculated that coarse-binned data contributed to the past discussion of compartments as largely multi-megabase features, within a hierarchical model (Rowley and Corces, 2018). Only a few years ago was it demonstrated that compartments represent a fairly independent structure from CTCF loops, and that alternating compartment intervals are often smaller than CTCF loop domains (Nora et al., 2017; Rao et al., 2017; Rowley et al., 2017). Indeed, small compartment intervals can even lie inside CTCF loop domains (Rowley et al., 2017; Gu et al., 2021).

Largely due to sequencing costs and experimental considerations, many Hi-C maps contain less than 1 billion read-pairs (Figure 5B). Indeed, sequencing depth has likely been a limiting factor for analysis considering the exponential relationship between 2D matrix filling and bin size. Illustrating this issue, a simplistic calculation indicates that a 10-fold increase in 2D coverage requires approximately 100-fold more sequenced reads: $2D\;coverage=\frac{reads}{{bins}^{2}}$ (Figure 4). Thus compartment calling at higher resolutions has exponentially less data per bin. It is important to consider how sequencing depth impacts our understanding of compartments as ultra-deep sequencing reveals many new aspects of fine-scale compartmental organization (Gu et al., 2021). The increasing ability to use machine learning to impute high-resolution data from maps with low sequencing depth may also help in this regard (Zhang et al., 2018; Carron et al., 2019; Liu et al., 2019; Liu and Wang, 2019; Schwessinger et al., 2020; Cheng et al., 2021; Tan et al., 2023). As the cost of sequencing decreases and deeply sequenced Hi-C and Micro-C maps become more common, it will be valuable determine the effectiveness of these imputation methods at fine-scale.

### 5.2 Data normalization

**FIGURE 6:**
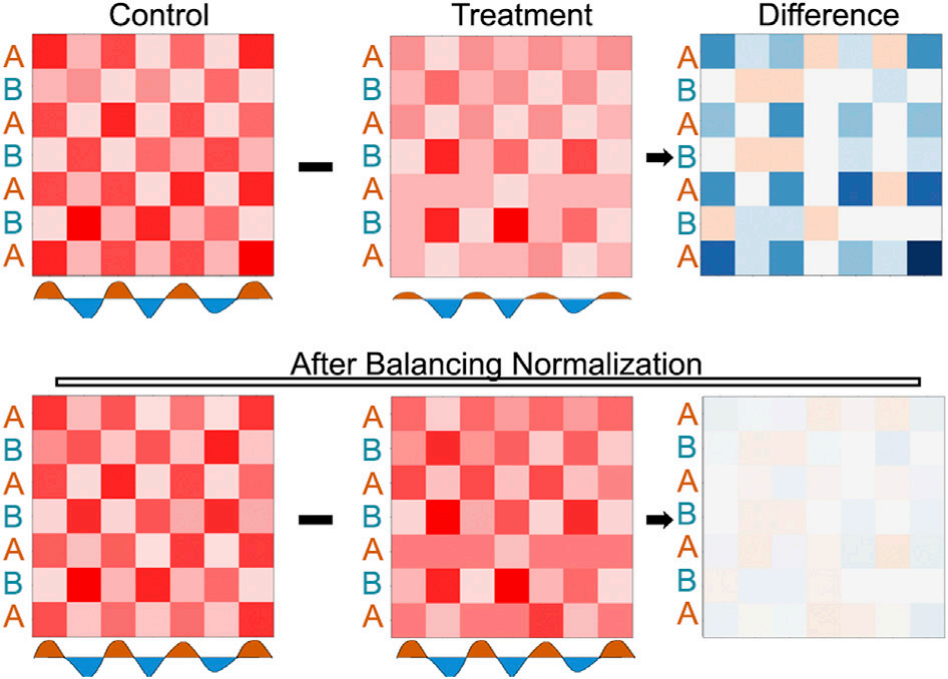
Demonstration of how matrix balancing can obscure widespread changes. Top row: An example checkerboard matrix (control) where the signal along the A compartment becomes decreased (treatment), the differences shown by the intensity of blue signal on the right (difference). Bottom row: The same checkerboards and the differences after matrix balancing.

In 3D genome contact maps, there are several factors that can lead to inherent visibility bias (Lieberman-Aiden et al., 2009; Yaffe and Tanay, 2011), and a typical workflow will try to normalize for these effects (Figure 2). However, normalization schemes make assumptions which should be considered during the analysis. For a simple example, coverage normalization assumes that each bin has the same potential to form interactions as every other bin; albeit with the consideration that some bins may interact more randomly than others. Interestingly, one study examined six different Hi-C normalization strategies, comparing them in several metrics, including their impact on visual quality, replicate comparison at various resolutions, consistency of the distance stratum, and TAD identification (Lyu et al., 2020). Each normalization scheme has its own advantages and disadvantages, and caution should be taken to avoid normalizations that may impact the interpretation of the data as it pertains to the specific research question. To illustrate how normalization may impact data analysis, we can imagine a matrix displaying a checkerboard pattern of signal (Figure 6) along with a hypothetical treatment that depletes all interactions in one compartment (i.e., the A compartment) (Figure 6, Treatment). In this scenario, differences between the matrices are readily evident without normalization (Figure 6, top row). However, these differences are muted after matrix balancing (Figure 6, bottom row). This extreme illustration serves as an example of how matrix normalization can obscure differences, but a counterargument is that avoiding the false detection of differences due to artificial visibility biases may be worth the cost of missing actual widespread changes. It is worth considering how the assumptions of normalization schemes may influence the interpretation of experimental results, particularly for compartmental interaction patterns that are spread across the entire chromosome.

### 5.3 Categorization vs. quantification of compartment states

**FIGURE 7:**
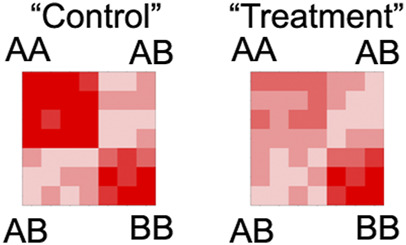
Illustrative example of how saddle plot analysis can detect changes in compartment interaction intensity. The example matrices from Figure 6 were used to demonstrate how resorting interactions can provide a useful visualization.

Compartment states are often analyzed as categorical features, e.g., intervals either belong to A or B. It is fairly common to report changes in compartments as the number of loci that switch from A to B or *vice versa*. While this simplification is useful to describe a dramatic and complete switch, it lacks quantitative power to examine changes to the intensity of compartmental association. Illustrating this point, our simple example demonstrates that large differences could be missed by categorical analysis (Figure 6) To overcome this challenge, many perform separate measurements of compartment interactions, such as comparing within v.s. between compartment states, e.g., $\frac{AA+BB}{{AB}^{2}}$ . Interactions can also be sorted by the eigenvector for a saddle plot analysis (Kruse et al., 2020; van der Weide et al., 2021; Magnitov et al., 2022) (Figure 7). While useful to independently measure the segregation of candidate loci, these metrics typically do not assign statistical significance to differential loci. Surprisingly, there are relatively few algorithms to statistically identify differential compartment intervals. One recently developed algorithm, dcHiC, compares quantile normalized eigenvectors, using the Mahalanobis distance with chi-square tests and *p*-value correction to assign statistical significance (Chakraborty et al., 2022). This method provides a statistical test to identify significantly differential compartment intervals between maps. Importantly, quantitative analyses provide mechanistic insights that could be missed by categorical approaches. In the future, implementing statistical measurements for differential interaction analysis will be essential to ensure robust and quantitative interrogation of compartments.

## 6 Discussion

Chromatin compartments are a widespread and prominent feature of chromatin organization, seen by chromatin conformation capture methods like Hi-C and Micro-C, non-ligation methods like Genome Architecture Mapping (GAM) and Split-Pool Recognition of Interactions by Tag Extension (SPRITE), by immunoprecipitation-based methods like HiChIP and ChIA-PET, and by high-resolution imaging like OligoStorm (Lieberman-Aiden et al., 2009; Fullwood et al., 2010; Mumbach et al., 2016; Beagrie et al., 2017; Rowley et al., 2017; Nir et al., 2018; Quinodoz et al., 2018; Hsieh et al., 2020; Krietenstein et al., 2020). While we focused on the identification of A/B compartments and sub-compartments from Hi-C and Micro-C, other methods have used the same or similar algorithms. For example, the eigenvector was recently used to identify A/B compartments in High throughput Pore-C (HiPore-C) (Deshpande et al., 2022; Zhong et al., 2023) data as well as in data from GAM and SPRITE, which are both non-ligation based methods (Beagrie et al., 2017; Quinodoz et al., 2018). Therefore, while current strategies have been exceedingly useful among multiple methods and propelled our knowledge of genome organization, looking to the future, we have suggested several aspects of compartmental analysis that should be considered, from data preparation steps to identification methods. Even common steps, such as data normalization and choice of bin-size, vary between studies and could lead to alternate findings. Considering these aspects ensures that the data preparation and analysis fit with the experimental question. These factors are particularly relevant to experiments that explore differential genome organization, as considering the limitations of an approach helps to ensure that actual differences are not missed by factors such as sequencing depth, coarse binning, matrix normalization, or inherent issues with categorization v. s. quantification. As our understanding of compartmental organization progresses, it will be important to evaluate the impact of these aspects more closely.

## 7 Tools and terminologies (alphabetical)

Calder (Liu et al., 2021)—A tool for calling subcompartments using hierarchical clustering. Uses intra-chromosomal contacts.

Chromatin Compartment—The organization and segregation of genomic loci into distinct chromatin states.

CoRNN (Zheng et al., 2022)—Compartment prediction using Recurrent Neural Network is a tool for predicting A/B compartments from histone modifications.

CScoreTool (Zheng and Zheng, 2018)—A tool for calculating A/B compartment intervals using iterative parameter tuning by maximum-likelihood estimation.

dcHiC (Chakraborty et al., 2022)—differential compartment analysis of Hi-C is a method for statistical testing of compartment differences.

GAM (Beagrie et al., 2017)—Genome Architecture Mapping provides chromosome contact maps by laser microdissection and sequencing.

GRO-seq (Core et al., 2014)—Global Run On Sequencing data provides a genome-wide measurement of active transcription.

H3K9me3—Histone 3 Lysine 9 tri-methylation is a chromatin modification typically associated with heterochromatin.

H3K27ac—Histone 3 Lysine 27 acetylation is a chromatin modification typically associated with active chromatin, particularly at active regulatory elements.

H3K27me3—Histone 3 Lysine 27 tri-methylation is a chromatin modification typically associated with silenced/repressive chromatin.

H3K36me3—Histone 3 Lysine 36 tri-methylation is a chromatin modification typically associated with the bodies of transcribed genes.

Hi-C (Lieberman-Aiden et al., 2009)—A genome-wide method of measuring chromatin contact maps by restriction enzyme digestion followed by ligation and sequencing.

HiChIP and ChIA-PET (Fullwood et al., 2010; Mumbach et al., 2016)—Methods of immunoprecipitating chromatin interactions bound by proteins of interest.

HiPore-C and Pore-C (Deshpande et al., 2022; Zhong et al., 2023)—A chromatin conformation capture method using Nanopore sequencing to enable identification of multi-way contacts.

ICE and KR (Imakaev et al., 2012; Knight and Daniel, 2013; Rao et al., 2014)—Iterative Correction and Eigenvector decomposition and Knight-Ruiz matrix balancing are popular normalization schemes for Hi-C matrices.

Micro-C (Hsieh et al., 2015; Hsieh et al., 2020; Krietenstein et al., 2020)—A genome-wide method of measuring chromatin contact maps by Micrococcal Nuclease digestion followed by ligation and sequencing.

MiChroM (Di Pierro et al., 2017)—Minimal Chromatin Model is a tool to predict compartments from ChIP-seq data within an energy landscape model of chromatin structure.

MOSAIC (Wen et al., 2022)—Modularity and Singular vAlue decomposition-based Identification of Compartments is a tool for calling subcompartments using clustering of PCA eigenvectors. Uses intra-chromosomal contacts.

OligoSTORM (Beliveau et al., 2017; Nir et al., 2018)—Oligopaint with Stochastic Optical Reconstruction Microscopy is a high-resolution microscopy-based method of measuring chromatin organization at individual loci which has been used to walk along compartmental domains.

PCA (Principal Component Analysis) leading eigenvector—A vector corresponding to the axis which captures the largest amount of variance in the data, often used to define A/B compartments from genome-wide maps of chromatin conformation.

POSSUMM (Gu et al., 2021)—PCA of Sparse SUper Massive Matrices is a tool for calculating the compartmental eigenvector from large matrices in sparse format.

SCI (Ashoor et al., 2020)—Sub-Compartment Identifier is a tool for calling subcompartments using graph embedding and k-means clustering. Uses inter-chromosomal contacts.

SNIPER (Xiong and Ma, 2019)—Subcompartment iNference using Imputed Probabilistic ExpRessions is a tool for calling subcompartments using a neural network. Uses inter-chromosomal contacts.

SPRITE (Quinodoz et al., 2018) - Split-Pool Recognition of Interactions by Tag Extension provides chromosome contact maps by split-and-pool barcoding. See also RNA and DNA (RD-) SPRITE which measures the organization of RNA and DNA (Quinodoz et al., 2021).
